# Supplementary material for: Effects of wind speed and wind direction on crop yield forecasting using dynamic time warping and an ensembled learning model
Source: PeerJ. 2024 Jun 11;12:e16538. doi: 10.7717/peerj.16538 (PMC11177857; doi:10.7717/peerj.16538)
Supplement: Supplemental Information 1 [file peerj-12-16538-s001.docx]

**Supplementary Sheet**

**Table 1. Model: Combined LSTM-DTW-RF Model**

| **Layer (type)** | **Output Shape** | **Param #** |
| --- | --- | --- |
| LSTM (LSTM) | (None, 50) | 10400 |
| DTW (Custom Layer | (None, ...) | ... |
| Random Forest | (None, 1) | 303 |
| **Total params:**  **Trainable params:**  **Non-trainable params:** | 10,703  10,703  0 |  |

**Table 2. DTW Model (Custom layer)**

| **Parameter** | **Description** |
| --- | --- |
| Window Size (W) | 2 |
| Window Type | Sakeochiba |
| Distance Metric | fastDTW |
| Step Pattern (S) | method='symmetricP2 |
| Normalization (N) | (len(sequence1) + len(sequence2)) |

**Table 3. Model: Combined LSTM-RF Model**

| **Layer (type)** | **Output Shape** | **Param #** |
| --- | --- | --- |
| LSTM (LSTM) | (None, 50) | 10400 |
| Random Forest | (None, 1) | 303 |
| **Total params:**  **Trainable params:**  **Non-trainable params:** | 10,703  10,703  0 |  |

**Table 4. Augmented Dickey-Fuller (ADF) test on results level of Confidence**

| **Information** | **LSTM-DTW-RF Value** | **LSTM-RF Value** |
| --- | --- | --- |
| Test Statistic | -6.022060 | -6.02206 |
| *p*-value | 1.4028797e-07 | 1.3153912e-01 |
| #Lags Used | 425 | 425 |
| Number of Observations Used | 517 | 517 |
| Critical Value (1%) | -3.435052e+00 | -3.175137e+00 |
| Critical Value (5%) | -2.861150e+00 | -260146e+00 |
| Critical Value (10%) | -2.603 | 1.827 |
| Level of Confidence | 90% | 87.99% |
| dtype | float64 |  |

**Breakdown of the sequential splitting for Time Series Cross-validation**

- **Split 1:** Training data from index 0 to 314, testing data from index 315 to 449
- **Split 2:** Training data from index 0 to 448, testing data from index 315 to 449, and index 0 to 67
- **Split 3:** Training data from index 0 to 448, testing data from index 68 to 201
- **Split 4:** Training data from index 0 to 448, testing data from index 202 to 335
- **Split 5:** Training data from index 0 to 448, testing data from index 336 to 449, and index 0 to 69
- **Split 6:** Training data from index 0 to 448, testing data from index 70 to 203
- **Split 7:** Training data from index 0 to 448, testing data from index 204 to 337
